# From dysbiosis to dysfunction: specific gut microbes and metabolites in the pathogenesis of Parkinson’s disease

**DOI:** 10.1093/sumbio/qvag002

**Published:** 2026-01-10

**Authors:** Fang Chi, Kun Chen, Yue Yin, Janetta R Hakovirta, Per E J Saris

**Affiliations:** Department of Microbiology, Faculty of Agriculture and Forestry, University of Helsinki, FI-00014 University of Helsinki, Finland; College of Food Science and Nutritional Engineering, China Agricultural University, 100083, Beijing, China; National Engineering Research Center for Fruit and Vegetable Processing, Key Lab of Fruit and Vegetable Processing, Ministry of Agriculture, Beijing Key Laboratory of Food Non-Thermal Processing, 100083, Beijing, China; Department of Microbiology, Faculty of Agriculture and Forestry, University of Helsinki, FI-00014 University of Helsinki, Finland; Department of Microbiology, Faculty of Agriculture and Forestry, University of Helsinki, FI-00014 University of Helsinki, Finland; Department of Microbiology, Faculty of Agriculture and Forestry, University of Helsinki, FI-00014 University of Helsinki, Finland

**Keywords:** Parkinson’s disease, gut microbes, microbial metabolites, microbiota-gut-brain axis, species-level microbes, microbiota-based therapy

## Abstract

Parkinson’s disease (PD) involves a complex interplay between the gut microbiota, their metabolites, and host neurophysiology. Studies across independent cohorts have begun to reveal reproducible microbial signatures, with taxa such as *Desulfovibrio* spp., *Akkermansia*, and *Bifidobacterium* repeatedly enriched, whereas Prevotellaceae and *Faecalibacterium* are consistently reduced. Beyond these broad compositional patterns, several species and strains—including *Helicobacter pylori*, curli-producing *Escherichia coli*, and *Desulfovibrio* spp.—have been linked to processes such as α-synuclein aggregation, immune activation, and dopaminergic vulnerability. Microbial metabolites including short-chain fatty acids, hydrogen sulfide, lipopolysaccharides, bile acids, and iron-related compounds provide additional mechanistic connections, influencing gut barrier function, inflammatory responses, and neuronal homeostasis. In this review, we bring together findings from taxonomic, metabolic, and mechanistic studies, evaluate the therapeutic potential of microbiota-targeted interventions. Future research should pivot from descriptive microbiome profiling toward mechanistic studies that delineate causal relationships between defined microbes, their metabolites, and PD pathology. Such efforts are essential for identifying early diagnostic biomarkers and developing targeted microbiota-based therapies that could alter the clinical course of PD.

**Sustainability Statement**

This review contributes to UN Sustainable Development Goal (SDG) 3, Good Health and Well-being, by synthesizing current knowledge on how gut microbiota influence Parkinson’s disease (PD) pathogenesis. By consolidating mechanistic insights into microbial dysbiosis, host responses, and neurodegeneration, it emphasizes the role of fundamental microbiome research in shaping our understanding of PD. Such basic research provides an essential scientific foundation for future strategies aimed at disease prevention, patient care, and long-term improvements in human health. This work also provides a foundation for future microbiome-based healthcare innovations.

## Introduction

Parkinson’s disease (PD) is the second most common neurodegenerative disorder after Alzheimer’s disease, affecting millions worldwide (Prymaczok 2024, Su 2025). The World Health Organization (WHO) reports a doubling of the global prevalence of PD in the past 25 years, over 8.5 million individuals lived with PD in 2019 alone (Organization 2022). In 2017, PD was estimated to incur annual costs of approximately $52 billion in the United States alone, a burden expected to rise further as both incidence and prevalence continue to increase (Yang 2020). By 2040, the number of individuals affected by PD is projected to exceed 14 million (Hiller 2022). Clinically, PD is characterized by motor symptoms including bradykinesia, rigidity, resting tremor, and postural instability (Hoglinger 2024, Morris 2024), as well as non-motor manifestations including gastrointestinal dysfunction (Hopfner 2017, Murros 2021, Yang 2023, Shih 2024). Pathologically, PD is defined by the degeneration of dopaminergic neurons in the substantia nigra pars compacta (Lakhani 2024), and the accumulation of misfolded alpha synuclein (α-syn aggregates) into Lewy bodies—a hallmark feature in disease diagnosis and pathogenesis (Morris 2024, Yan 2024). Although the exact PD etiology remains multifactorial, emerging evidence implicates dysregulated calcium homeostasis, endoplasmic reticulum stress (Wang 2023a, Cali 2011 et al. 2011), mitochondrial dysfunction (Kaur 2023, Mnich 2023, Sohrabi 2023), oxidative stress (Chakrabarti and Bisaglia 2023, Yuan 2024), and neuroinflammation as key drivers (Kouli 2024). Gastrointestinal (GI) dysfunction is now recognized as a pivotal factor in PD’s pathogenesis (Mukherjee 2016 et al. 2016), suggesting involvement of the gut-brain axis in the initiation of PD (Hopfner 2017, Murros 2021, Zhang 2023, Shih 2024).

Clinical studies have documented GI abnormalities in PD patients, including weight loss, gastroparesis, constipation, and defecatory dysfunction (Sun and Shen 2018, Dodiya 2020, Zhang 2023). Impaired gut barrier integrity, increased intestinal permeability, and chronic low-grade inflammation have also been widely documented in PD (Kishimoto 2019, Perez-Pardo 2019, van and Derkinderen 2019, Yang 2022). GI dysfunction may serve as a pathway for pathology to spread from the enteric nervous system (ENS) to the central nervous system (CNS) (Travagli 2020 et al. 2020, Metta 2022, Warnecke 2022). This gut-origin hypothesis is supported by pathophysiological evidence that α-syn inclusions first appear in the ENS and later propagate to the brain via the vagus and glossopharyngeal nerves (Bottner 2012, Chalazonitis and Rao 2018, Manfredsson 2018, Yang 2023, d’Angelo 2024, Espinosa-Oliva 2024). The ENS, often termed the ‘second brain,’ comprises of an autonomous neural network of neurons and glia embedded in the gut wall. It communicates through diverse neurotransmitters and operates semi-autonomously from the CNS to regulate gut motility, secretion, and immune responses (Viola 2023). The gut microbiota, comprising of trillions of bacteria, influences bidirectional gut-brain communication through immune, endocrine, and neural pathways—mechanisms implicated in PD (Turnbaugh 2006). Undoubtedly, research on the gut, gut microbiota, and PD has attracted much attention.

**Figure 1:**
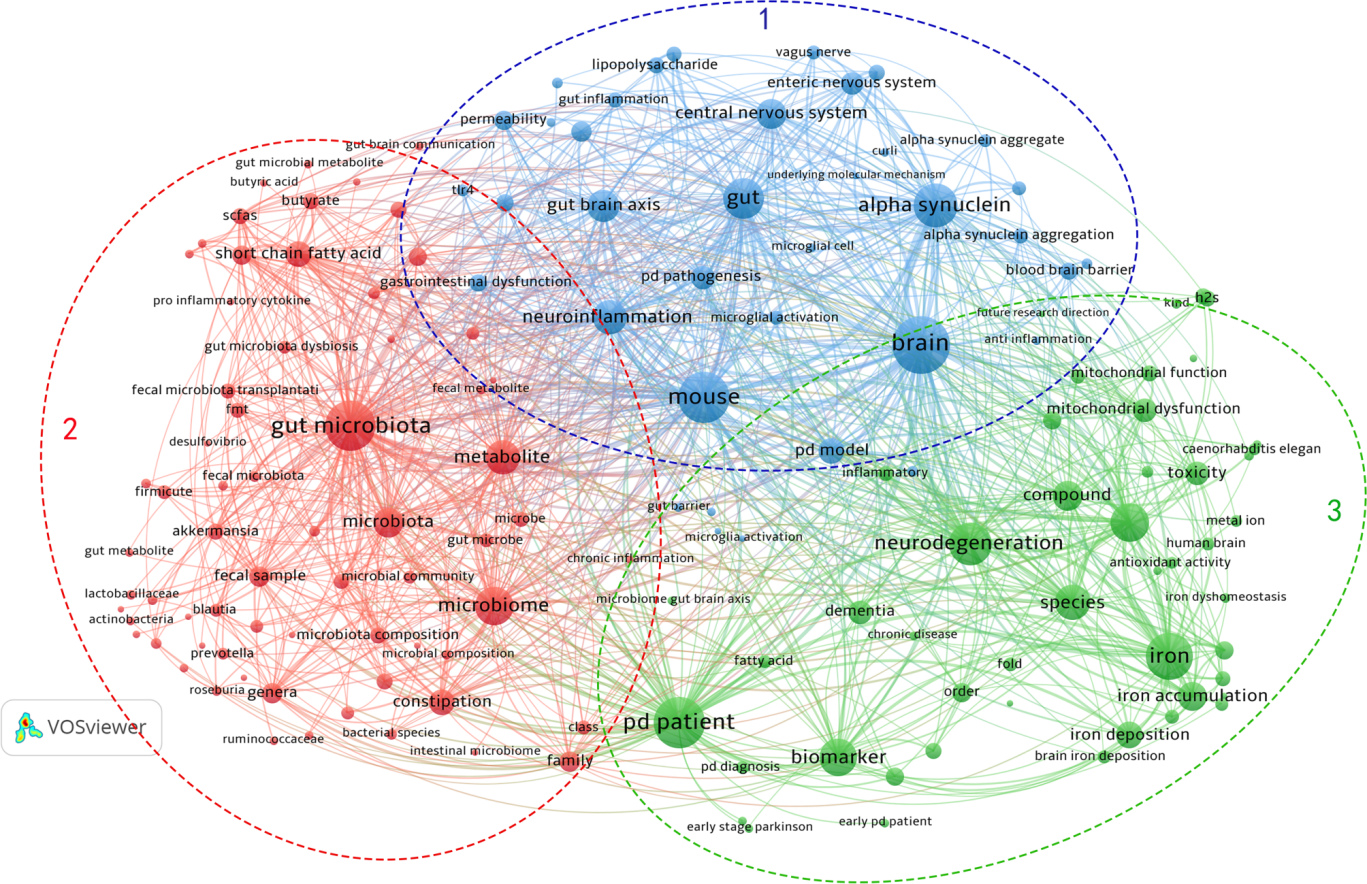
Keyword co-occurrence network of gut microbiota–Parkinson’s disease research. Overlay visualization of keyword co-occurrence from the titles and abstracts of 240 references included in this review. Nodes represent keywords, with node size indicating term frequency and connecting lines denoting co-occurrence relationships. The network was generated using VOSviewer (version 1.6.20; Leiden University, The Netherlands).

Nowadays, many researchers pay great attention to the effects of gut microbiota on PD (Wu 2025b, Scheperjans 2015, Sampson 2016, Ni 2025), particularly their association with gut microbiota alterations (Fig. 1). The three clusters reflect a multidimensional research landscape connecting gut microbiota composition, microbial metabolism, and PD pathogenesis at both mechanistic and clinical levels. Cluster 1 (blue), focused on intestinal and animal models in PD research, this cluster includes terms such as gut, brain, mouse, alpha-synuclein, gut–brain axis, inflammatory bowel disease, enteric nervous system, and vagus nerve. The most frequent term in this cluster is brain, followed by gut, mouse, and alpha-synuclein. The layout suggests that gut serves as a central node connected to PD through the brain, highlighting the importance of gut–brain interactions in PD pathogenesis. Cluster 2 (red), centered on gut microbiota—the most frequent keyword in the dataset—this cluster includes microbiome, metabolite, constipation, fecal sample, short-chain fatty acid, and butyrate. This cluster underscores research emphasizing taxonomic shifts and microbial metabolite production in PD. Cluster 3 (green), the largest cluster, comprising 30 keywords, centers on PD pathology. Key terms include alpha-synuclein, oxidative stress, mitochondrial dysfunction, iron accumulation, biomarker, and early diagnosis. This group reflects efforts to link molecular mechanisms of neurodegeneration to clinical translation, including biomarker discovery and therapeutic targeting. Typically, PD patients exhibit distinct gut microbiome signatures, characterized by reduced microbial diversity. For example altered Firmicutes/Bacteroidetes ratios, and shifts in metabolite profiles (e.g. short-chain fatty acids, lipopolysaccharide) (Lin 2018, Murros 2021, Tran and Mohajeri 2021, Zheng 2021, Warnecke 2022). The abundance of *Prevotellaceae* is reduced in the feces of PD patients (Wang 2024b). However, increased levels of *Enterobacteriaceae* have been positively associated with postural instability and gait difficulty in PD (Scheperjans 2015). In summary, dysbiosis of the gut microbiota has been widely linked to PD.

Functioning as a metabolic bioreactor, the gut microbiota exhibits remarkable enzymatic capacity, generating a wide array of bioactive compounds that modulate host physiology (Chen 2019b, Rooks and Garrett 2016) and contribute to immune regulation, nutrient metabolism, and neuroimmune signaling (Fischbach and Segre 2016, Fan and Pedersen 2021, Murros 2021). The microbiota-derived metabolites can act locally within the intestinal lumen or enter the systemic circulation where they exert functional effects on distant organs and tissues (Turnbaugh 2006, Rooks and Garrett 2016, Morais 2021 et al. 2021, Murros 2021). For example, SCFAs (Yang 2022), hydrogen sulfide (Murros 2022), amino acid metabolites, bacterial amyloids, and iron compounds—serve as signaling molecules that activate the vagus nerve, which then modulates both central microglial activity and peripheral immune responses (Zhao 2019b, Camara 2015, Kaczmarczyk 2017). The observed depletion of fecal SCFAs, which are known to maintain epithelial barrier function, may contribute to intestinal barrier disruption, leading to increased epithelial permeability and the initiation of local inflammatory responses (Stevens 2018, Zhao 2018, Stephens and von der Weid 2020, Vicentini 2021). Increased intestinal permeability allows microbial metabolites and cytokines to translocate into systemic circulation, promoting chronic low-grade inflammation and compromising the integrity of the blood-brain barrier (Tran and Mohajeri 2021, Yang 2022). This cascade facilitates the entry of pro-inflammatory mediators into the brain, where they can activate microglia and trigger neuroinflammatory responses (Nighot 2019, Dumitrescu 2021, Zhou 2022). Above all, these metabolites act as key mediators of microbiota–host interactions, exerting wide-ranging effects on host physiology. They can modulate immune responses and influence intestinal barrier integrity, either reinforcing mucosal protection or exacerbating gut permeability (Sun and Shen 2018, van Kessel and El Aidy 2019, Zhou 2019, Liang 2021).

Although the gut microbiota has gained considerable attention as a potential contributor to PD pathogenesis, the precise interrelationship between PD and gut microbiota remains unclear. Most current investigations rely heavily on sequencing-based approaches to characterize taxonomic shifts in PD-associated microbiota, primarily at the phylum, family or genus level. The functional implications of specific microbial taxa and their metabolites remain poorly understood, limiting progress toward mechanistic insight, early diagnostic markers, and microbiota-targeted therapies (Fan and Pedersen 2021, Cannon and Gruenheid 2022, Deng 2024). To address this gap, this review transitions from macro- to micro-level perspectives. First, we summarize compositional changes in gut microbiota across major taxonomic ranks and identify consistent trends observed in PD. Next, we focus on specific microbial taxa *Escherichia coli, Helicobacter pylori*, and *Desulfovibrio* spp.—that are repeatedly implicated in PD pathophysiology. Then, we explore microbial metabolites including SCFAs, lipopolysaccharide (LPS), curli fibers, and bile acids, emphasizing their potential roles in barrier integrity, neuroinflammation, and α-syn aggregation. Additionally, current therapeutic approaches targeting the gut microbiota—including antibiotics, probiotics, and fecal microbiota transplantation (FMT)—are evaluated for their potential clinical value. By integrating lessons from microbiome research in other human diseases, we propose future directions and conceptual frameworks for advancing PD-microbiota studies. By bridging compositional data with functional insights, this review aims to contribute to a mechanistic understanding of gut microbiota-PD interactions and to inspire microbiota-targeted strategies for early diagnosis and neuroprotection.

### Alterations in gut microbiota composition in Parkinson’s disease

**Figure 2:**
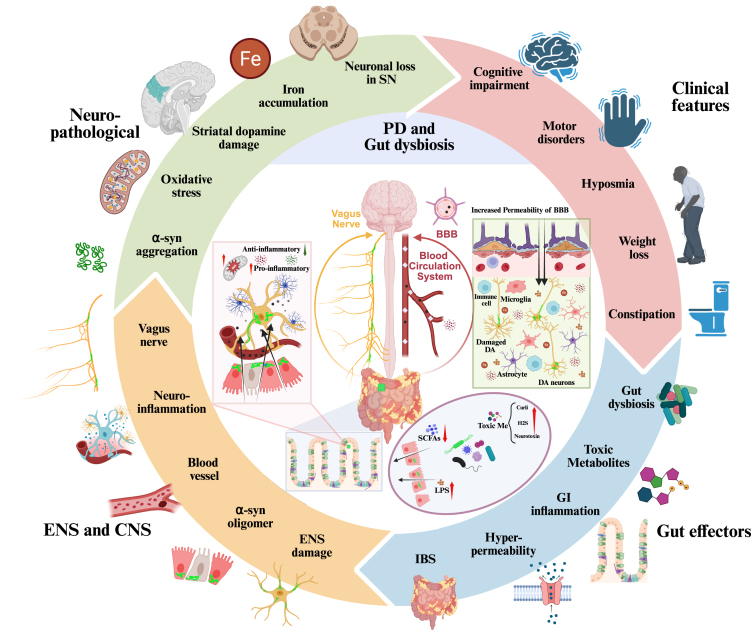
The cyclical and bidirectional interplay between gut dysbiosis and PD pathology via the microbiota-gut–brain axis. Gut effectors & dysbiosis (lower right). Propagation via ENS & CNS (lower left). Bidirectional propagation to the brain (central insets), including vagus nerve transmission and blood brain barrier (BBB).Neuropathological cascades (upper left) . Clinical features (upper right) . This figure was created with BioRender.com under an institutional license provided by the University of Helsinki.

PD is not confined to the brain but is closely linked to the gut (Fig. 1 and 2), where microbiota dysbiosis and altered microbial metabolites form an interconnected system influencing neurodegeneration (Sampson 2016, Aho 2019). At the phylum level, Bacteroidetes, Proteobacteria, and Verrucomicrobia are elevated in PD patients, correlating positively with disease (Keshavarzian 2015), whereas Firmicutes, Tenericutes, and Euryarchaeota are significantly reduced (Lin 2018). At the family level, Prevotellaceae abundance is reduced by over 77% in fecal samples from PD patients, and its depletion has been linked to reduced mucin synthesis and increased intestinal permeability. The relative abundance of Enterobacteriaceae was positively associated with the severity of postural instability and gait difficulty (Scheperjans 2015). Other families such as Coprobacillaceae and Lachnospiraceae are depleted (Keshavarzian 2015), while Bifidobacteriaceae, Ruminococcaceae, Verrucomicrobiaceae, and Christensenellaceae are enriched and associated with disease duration (Hill-Burns 2017, Aho 2021, Shen 2021). The observed decrease in Prevotellaceae abundance aligns with findings of increased gut permeability in PD, as low levels of *Prevotella* may reduce mucin synthesis, a factor associated with heightened gut permeability (Brown 2011, Forsyth 2011, Aho 2021). Research from China indicates that the abundance of Lachnospiraceae was reduced by 42.9% in patients with PD, while Desulfovibrionacea and Bifidobacteriaceae were found to be enriched in PD patients (Lin 2018).

At the taxonomic genus level, several putative anti-inflammatory, butyrate-producing taxa—including *Blautia, Coprococcus*, and *Roseburia*—have been consistently reported at higher abundances in healthy controls compared to patients with PD. In contrast, *Dorea* and *Faecalibacterium*, two genera involved in SCFA production, are significantly decreased in the mucosa of PD patients, with levels of *Blautia* and *Faecalibacterium* negatively correlating with disease duration (Keshavarzian 2015). Among the most consistent gut microbiome signatures in PD are the enrichment of *Lactobacillus, Akkermansia*, and *Bifidobacterium*, alongside the depletion of *Faecalibacterium* (Hasegawa 2015, Keshavarzian 2015, Unger 2016, Hill-Burns 2017). SCFAs such as butyrate are considered beneficial microbial metabolites due to their ability to support gut mucosal integrity and induce anti-inflammatory cytokine expression (Sokol 2008, Hirayama and Ohno 2021). The loss of SCFA-producing genera including *Blautia, Coprococcus*, and *Roseburia* has been widely observed in PD patients (Keshavarzian 2015). Conversely, putative “pro-inflammatory” bacteria including Oxalobacteraceae and *Ralstonia* exhibited significantly higher abundance in the mucosa of PD patients than controls (Shi 2023).

At the species level, two independent studies have reported the same conclusion: a noteworthy correlation between *Desulfovibrio* (DSV) and PD. Significantly elevated levels of DSV is observed in the fecal samples of PD patients (Murros 2021, Nie 2023), and DSV abundance was positively correlated with disease severity. DSV is classified as a sulfate-reducing bacterium (SRB) capable of inducing infections and producing hydrogen sulfide (H₂S)—a microbial metabolite known to modulate neuronal cell signaling even at low concentrations, but highly toxic at elevated levels (Ijssennagger 2016 et al. 2016, Buret 2022). Also, H_2_S can trigger the release of cytochrome c from mitochondria, which is considered the initial step in alpha-synuclein aggregation. This visualization highlights the multi-level taxonomic shifts characterizing PD gut dysbiosis and underscores the convergence of findings across independent studies.

Gut microbial dysbiosis is not restricted to humans and has also been consistently observed across multiple animal models of PD (Sampson 2016, Bhattarai 2021). Germ-free mice exhibit widespread microglial abnormalities, including altered morphology and impaired maturation, which collectively impair innate immune responses (Erny 2015, Madore 2020). Conversely, reintroducing a complex microbiota or administering microbial metabolites—particularly a cocktail of SCFAs—restores microglial density, composition, and surface marker expression, thereby ameliorating microglial deficiencies (Erny 2015, Mishra 2023). In rotenone-induced PD mouse models, the genera *Desulfovibrio, Helicobacter*, and *Akkermansia* were significantly enriched in fecal samples. These alterations correlated with behavioral impairments, gastrointestinal dysfunction, and inflammatory markers (Zhao 2021). The enrichment of *Desulfovibrio* was consistent with findings from human PD cohorts. In a separate study, alterations in the colonic microbiota of PD mice closely mirrored human findings, including increased Verrucomicrobiaceae and decreased Prevotellaceae abundance (Song 2020). In an MPTP-induced PD mouse model, gut microbiota composition was significantly altered, with significant changes in Lachnospiraceae, Erysipelotrichaceae, Prevotellaceae, Clostridiales, Erysipelotrichales, and Proteobacteria (Li 2020b). In PD mouse models, pathological α-syn fibrils injected into the duodenal and pyloric muscularis layers propagated in a stereotyped manner from the gut to the brain via the vagus nerve. Strikingly, truncal vagotomy or α-syn deficiency completely blocked gut-to-brain propagation of α-syn pathology, thereby preventing associated neurodegeneration and behavioral impairments (Kim 2019b). Collectively, findings from both human PD cohorts and animal models consistently reveal gut microbial alterations associated with PD.

**Figure 3:**
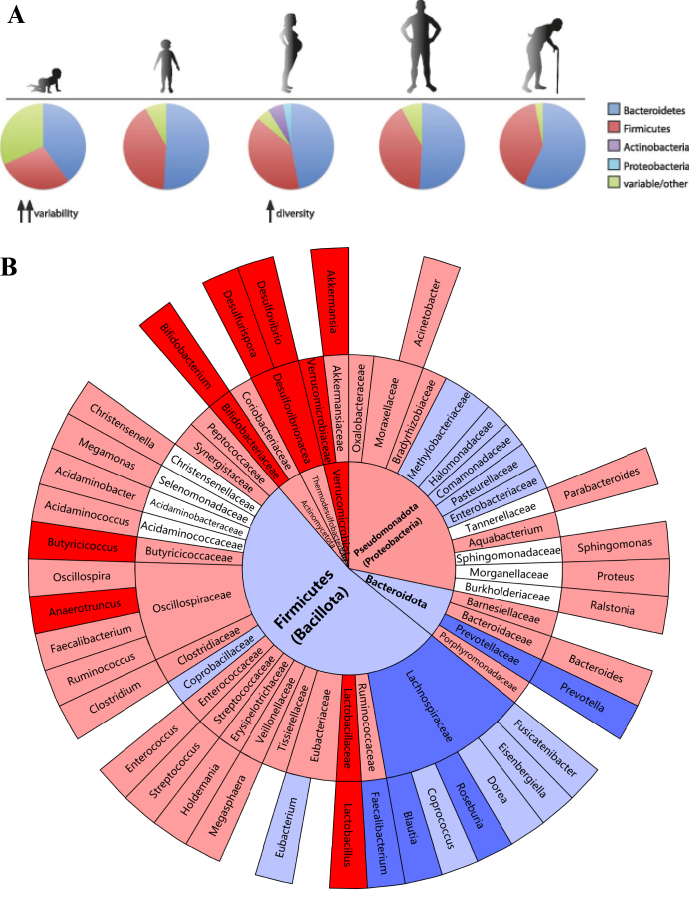
Altered gut microbiota structure in individuals with PD compared to healthy individuals. (A) The composition of the gut microbiota changes across the human lifespan (Kostic et al. 2013). (B) Differential bacterial taxa between PD and healthy individuals. Red means taxa significantly increased in PD and frequently reported, and pink means moderate increases. Blue menasans t significantly decreased and consistently replicated across cohorts, and purple showing moderate decreases.

It is crucial to note that various research outcomes may be influenced by sample characteristics, including gender, age, geographical location, lifestyle habits, diet, duration of PD, sample sources, and medication usage. Methodological differences—including sample storage conditions, DNA extraction protocols, sequencing platforms, bioinformatic pipelines, and taxonomic reference frameworks—further complicate cross-study comparisons. Despite this heterogeneity, several microbial families—including Desulfovibrionacea, Bifidobacteriaceae, Christensenellaceae, Ruminococcaceae, and Verrucomicrobiaceae—are consistently enriched in PD across multiple studies (Fig. 3). These taxa, along with their metabolic products, may contribute to host dysfunction and neurodegeneration. Conversely, reductions in Prevotellaceae, Lachnospiraceae, and Faecalibacterium—taxa often associated with anti-inflammatory and mucosal-protective functions—suggest a potential loss of beneficial microbial activity in PD. However, taxonomic shifts alone may be insufficient to fully explain how specific microbial communities contribute to PD onset and progression. Future studies should prioritize isolating candidate pathogenic microbes and elucidating their interactions with the enteric and central nervous systems. A mechanistic understanding of these relationships will be essential for advancing PD diagnostics and therapeutics. Collectively, evidence from both human and animal studies supports the concept that gut microbiota alterations are integral to PD pathogenesis, reinforcing the need to explore microbiota-targeted interventions as viable therapeutic strategies.

### Species and strain level pathogens as potential triggers in Parkinson’s disease

The impact of gut microbiota on PD cannot be fully understood from compositional shifts alone, moving from associations to causality requires species- and strain-level resolution (Lin 2018, Murros 2021, Nie 2023). While no microbial ‘smoking gun’ for PD exists, candidate pathogens emerge from experimental models through distinct mechanisms. In this section, we focus on species- and strain-level candidates that repeatedly emerge across cohorts and evaluate experimental and clinical evidence implicating them as potential drivers of PD pathogenesis. For instance, *Escherichia coli* (Liang 2023) and *Desulfovibrio* spp. (Huynh 2023) can promote α-syn misfolding and neuronal toxicity through direct mechanisms involving endotoxins (e.g. lipopolysaccharide) and oxidative stress (Boktor 2023). In contrast, *Clostridioides difficile* and *Helicobacter pylori* may contribute indirectly by disrupting gut barrier integrity, enhancing systemic inflammation, and priming neuroimmune responses (Shen 2017, Heils 2023). Importantly, these microbes may act synergistically as potential pathogens within a dysbiotic microbial community, with their relative abundance and metabolites, such as reduced short-chain fatty acids, increased LPS, curli amyloid protein and hydrogen sulfide production. Moving forward, establishing causality will require strain-resolved multi-omics approaches, such as metagenomics combined with spatial metabolomics and humanized gnotobiotic models, to bridge the gap between microbial associations and mechanistic insights into species- and strain-level pathogens PD. As *Helicobacter pylori* revolutionized our understanding of gastroduodenal diseases, is it conceivable that a comparable microbial driver—whether a single pathobiont or a pathogenic consortium—could similarly redefine the PD etiology? Answering this question would not only resolve longstanding debates about environmental triggers but also catalyze the development of microbiome-targeting neuroprotective strategies.

*H. pylori* infection has been associated with greater PD severity and reduced responsiveness to pharmacological treatment (Wang 2024a, Huang 2018, Fu 2019 et al. 2019, Zhong 2022). Epidemiological data from large-scale studies consistently show elevated *H. pylori* prevalence (32.4%) in PD cohorts compared to matched controls (Tan 2015, Shen 2017). This association remains statistically significant even after adjusting for potential confounding factors such as age, socioeconomic status, and medication use (Lee 2008, Wei 2024). A Mendelian randomization analysis reveals that, although *H. pylori* infection does not increase the risk of PD, it is implicated in the worsening of PD motor and cognitive symptoms (Wang 2024a). *H. pylori* eradication has been shown to improve motor fluctuations in PD patients (Lee 2008). These data suggest *H. pylori* as a disease-modifying factor in PD through dual impacts on symptom progression and treatment efficacy. *H. pylori* is a gram-negative, spiral-shaped, microaerophilic, and flagellated bacterium that colonizes the human gastric mucosa and infects approximately half of the global population (Li 2023, Malfertheiner 2023). It primarily inhabits the stomach, where it can cause chronic gastritis, peptic ulcers, and gastric cancer, and is classified as a Group I carcinogen by the WHO (Duan 2023). Beyond the gastrointestinal system, growing evidence suggests that *H. pylori* infection exerts systemic effects (de Korwin 2017, Park 2021), including neurological manifestations relevant to PD (Alvarez-Arellano and Maldonado-Bernal 2014, Kira and Isobe 2019, Gorlé 2021). These neurological effects are thought to arise via systemic inflammation (Tan 2015), disruption of gut–brain signaling, and the induction of neuroinflammatory cascades (McGee 2018 et al. 2018, Heiss and Olofsson 2019, Doulberis 2021). Several mechanistic hypotheses have been proposed to explain the *H. pylori*–PD association: (1) Inflammation by toxins and metabolites released by *H. pylori* stimulate the release of pro-inflammatory cytokines, promoting central inflammation and subsequent neuronal damage (Tan 2015, Shamsdin 2022, Wei 2024). (2) Altered drug pharmacokinetics by gastric colonization of *H. pylori* impairing levodopa absorption, potentially compromising its therapeutic efficacy and contributing to motor fluctuations (Pierantozzi 2006, Hashim 2014, Pfeiffer 2020 et al. 2020). (3) *H. pylori*-induced injury to the upper gastrointestinal mucosa may impair gut barrier function and facilitate PD-related pathogenesis (Chang 2024 et al. 2024). Collectively, these observations support a multifactorial connection between *H. pylori* infection and PD pathophysiology, underscoring the potential value of bacterial eradication in alleviating motor symptoms and modulating disease progression.

*Desulfovibrio* species have consistently emerged as microbial signatures associated with PD (Nie 2022, Singh 2023). Quantitative analyses indicate a significantly increased abundance of *Desulfovibrio* spp.—including *D. fairfieldensis, D. desulfuricans, D. piger*, and *D. vulgaris*—in the fecal microbiota of PD patients (3.3 × 10^7^ bacteria/g feces) relative to healthy controls (1.9 × 10^6^ bacteria/g feces) (Murros 2021, Huynh 2023). These findings have been independently replicated across geographically diverse cohorts, including studies conducted in China and Finland, and the abundance of *Desulfovibrio* has been positively correlated with PD symptom severity (Murros 2021, Nie 2023). In a *Caenorhabditis elegans* model of PD, exposure to PD patient’s *Desulfovibrio* strains promoted α-syn aggregation, both in terms of aggregate size and quantity (Huynh 2023), whereas *Desulfvibrio* strains from healthy did not. Interestingly, PD patient *Desulfovibrio* did not only trigger α-syn aggregation but also decreased expression of the proteostasis responsible for resolving α-syn aggregates (Mohammadi 2025 et al. 2025). The potential neurotoxicity of the PD patient’s *Desulfovibrio* strains bacteria is attributed to several metabolites, including lipopolysaccharide (LPS), hydrogen sulfide, and magnetite (Murros 2021, Murros 2022). These microbial products may promote α-syn misfolding, oligomerization, and aggregation—hallmark features of PD pathogenesis—either locally within the gut or following translocation to the central nervous system via the vagus nerve and systemic circulation. In addition, *Desulfovibrio*-derived metabolites and α-syn aggregates may compromise gut epithelial barrier integrity and trigger neuroinflammatory cascades, thereby exacerbating disease progression. Notably, *Desulfovibrio* enrichment is not unique to PD; similar patterns of dysbiosis have been reported in autism spectrum disorders (ASD), suggesting potential shared microbial pathways across neurodegenerative and neurodevelopmental conditions (Karnachuk 2021). Together, these findings position *Desulfovibrio* as a compelling candidate for microbial-driven α-synopathy and warrant further investigation into its mechanistic role in PD and related disorders.

*Escherichia coli* is a facultative anaerobic bacterium that typically exists as a commensal in the vertebrate gut but can also function as an opportunistic pathogen in mammals. Certain *E. coli* strains possess virulence factors that enable them to cause a wide range of intestinal and extraintestinal infections by disrupting multiple host cellular processes (Croxen and Finlay 2010, Denamur 2021). Some *E. coli* and other members of the *Enterobacteriaceae* family are capable of producing curli—extracellular amyloid fibers, that decorate the bacterial surface (Cegelski 2009). Curli fibers have garnered particular attention due to their amyloidogenic properties, which have been implicated in the pathogenesis of neurodegenerative and protein-misfolding disorders in humans (Chapman 2002, Barnhart and Chapman 2006). Experimental studies have shown that curli-producing *E. coli* strains promote α-syn aggregation and motor deficits in mice (Sampson 2020). Similarly, in *Caenorhabditis elegans* models expressing human α-syn, curli exposure significantly enhanced protein aggregation (Chen 2016, Huynh 2023). Mechanistically, curli fibers activate Toll-like receptor 2 (TLR2), triggering the release of pro-inflammatory mediators such as interleukin-8 (IL-8), interleukin-6 (IL-6), and tumor necrosis factor-α (TNF-α) (Tükel 2009, Tursi and Tukel 2018). These findings suggest that bacterial amyloid curli may act as a trigger for α-syn aggregation and a priming factor for inflammatory responses. Although curli-producing *E. coli* strains have been shown to promote α-syn pathology in both the gut and brain in preclinical models, a direct association between curli *E. coli* colonization and PD in humans remains unconfirmed. Future clinical investigations, leveraging large-scale microbiome datasets and strain-resolved metagenomics, are required to elucidate the potential role of curli *E. coli* in PD pathogenesis. These insights collectively emphasize the need to explore bacterial amyloids not merely as biomarkers, but as active molecular contributors to neurodegenerative cascades in PD.

Beyond the extensively studied taxa, emerging evidence suggests that *Proteus mirabilis* produces amyloid-like fibers capable of interacting with α-syn and promoting its aggregation. In mouse models, oral administration of *P. mirabilis* induces motor deficits and dopaminergic neuronal loss, accompanied by increased α-syn deposition in both the brain and colon (Choi 2018, Huh 2023). Similarly, *Clostridioides difficile* has been associated with an elevated short-term risk of PD (Kang 2020). In mice, *C. difficile* infection (CDI) alters dopamine metabolism in key dopaminergic brain regions, suggesting a possible mechanistic link between CDI and disruption of the dopaminergic axis (Vinithakumari 2022). While considerable attention has been directed toward individual microbial species implicated in PD—such as pro-inflammatory *Escherichia coli* or hydrogen sulfide-producing *Desulfovibrio* spp.—it is increasingly recognized that these organisms rarely act in isolation. Instead, their pathogenic potential is modulated, amplified, or suppressed by interactions within the broader microbial ecosystem. Microbe–microbe interactions may serve as critical amplifiers or attenuators of microbial pathogenicity. For instance, the deleterious effects of specific taxa can be exacerbated by the loss of beneficial commensals, such as the butyrate-producing *Faecalibacterium prausnitzii* or by the reduction of competitive species like *Bifidobacterium* spp.. A consistent shift toward a “low-SCFA, high-LPS” microbial profile in PD patients supports the hypothesis that ecological dysbiosis, rather than single-pathogen etiology, may underlie disease progression.

### Gut microbial metabolites as mechanistic mediators in Parkinson’s disease

Microbial metabolites are now recognized as active modulators of host physiology, shaping intestinal and central nervous system homeostasis through immune, endocrine, and neural pathways. Here, we focus on the major metabolite classes—short-chain fatty acids, lipopolysaccharides, hydrogen sulfide, bile acids, and iron-associated compounds—and highlight their mechanistic roles in PD pathogenesis (Chen 2022a,b, Sun 2018, Cirstea 2020, Vascellari 2020, Liang 2021, Yan 2021). The gut microbiota not only regulates gastrointestinal homeostasis—affecting motility, permeability, secretion, and immune responses—but also participate in bidirectional communication with both the enteric and central nervous systems via the microbiota–gut–brain axis (Torres-Fuentes 2017, Socala 2021). This integrative communication network functions through four primary pathways: (i) vagal nerve signaling, (ii) immune and inflammatory modulation, (iii) neuroendocrine regulation, and (iv) the production of microbial metabolites (Klingelhoefer and Reichmann 2015, Cryan 2019). Whether driven by intrinsic or extrinsic factors, dysbiosis alters the production of microbial metabolites in the gut. These microbial metabolites exert profound effects on neurotransmission, neuroinflammation, mitochondrial dynamics, and proteostasis, while CNS-derived signals reciprocally shape gut microbial composition and function. Microbial metabolites can access the CNS via systemic circulation and afferent neural routes, where they modulate neuromodulatory signaling, neuroinflammation, and neurodegeneration. Despite mounting insights, a fundamental gap persists in understanding how gut microbiota and their metabolites shape the progression of PD, regulate neurophysiological functions, and modulate disease severity. Emerging evidence suggests that microbial metabolites are key mediators of neuroinflammation, gut barrier disruption, mitochondrial dysfunction, and α-syn pathology—processes that collectively drive the trajectory of PD pathogenesis. The following sections provide a comprehensive overview of microbiota-derived metabolites implicated in PD, with a focus on elucidating their mechanistic roles in disease onset and progression.

Short-chain fatty acids (SCFAs), key metabolites produced by the gut microbiota, have garnered increasing attention for their roles in host–microbiome crosstalk and the pathophysiology of neurodegenerative diseases, including PD (Aho 2021, Kalyanaraman 2024 et al. 2024, Liu 2024). Perturbations in SCFA production have been linked to PD pathogenesis through modulation of peripheral immune activity and disruption of central nervous system homeostasis (Cao 2022, Yang 2022). SCFAs—acetate, propionate, and butyrate—are key metabolic byproducts produced by commensal gut bacteria via the fermentation of dietary fibers (Chen 2022a, Matt 2018, Vascellari 2020, Fock and Parnova 2023). SCFAs may migrate from the intestinal mucosa into the systemic circulation, where they can influence immune regulatory mechanisms and CNS functionality (Borre 2014, Erny 2015). These molecules play vital roles in maintaining gut homeostasis, modulating immune responses, and influencing the gut-brain axis.

SCFAs can modulate neuroinflammation by shaping the maturation, morphology, and function of microglia—the resident immune cells of the CNS (Caetano-Silva 2023). In a healthy state, SCFAs promote an anti-inflammatory microglial phenotype which is neuroprotective (Tedelind 2007). Gut dysbiosis often leads to altered SCFA production in PD (Unger 2016). Reduced butyrate levels have been associated with increased neuroinflammation (Matt 2018) and compromised intestinal barrier function, enabling the systemic translocation of pro-inflammatory mediators and their entry into the CNS (Qiao 2020, Aho 2021, Guo 2023). SCFAs also modulate the gut–brain axis via diverse routes, they activate the vagus nerve (VN)—a major conduit for bidirectional gut–brain communication (Goswami 2018 et al. 2018). Vagal activation has been associated with improved behavioral outcomes, preservation of neuronal populations, and reduced α-syn burden and inflammation in experimental PD models (Farrand 2017, Goswami 2018 et al. 2018, Farrand 2020). SCFAs can strengthen the integrity of the blood-brain barrier (BBB), and sodium butyrate has shown neuroprotective effects by restoring BBB function in murine models of brain injury (Li 2016, Fock and Parnova 2023). The depletion of SCFA-producing taxa such as Lachnospiraceae and *Faecalibacterium* genus reflects pronounced microbial dysbiosis in PD. This microbial imbalance may contribute to a pro-inflammatory milieu, potentially underpinning the chronic gastrointestinal symptoms observed in PD patients (Romano 2021). SCFA levels are reduced in the feces, but paradoxically elevated in the plasma of individuals with PD—potentially reflecting increased intestinal permeability (Galvez 2005 et al. 2005, Yang 2022). The cumulative evidence indicates SCFAs as critical modulators of gut barrier integrity, microglial activation, and gut–brain signaling, thereby offering mechanistic insights into the contribution of microbial metabolism to PD progression.

Lipopolysaccharide (LPS), a key structural component of the outer membrane of Gram-negative bacteria, has been increasingly implicated in the pathogenesis of PD (Sharma and Nehru 2015, Nighot 2019, Chen 2021, Zhao 2023). In PD, gut dysbiosis is often marked by an overrepresentation of gram-negative taxa, leading to elevated LPS production (Brown 2023 et al. 2023). Excess LPS disrupts epithelial tight junctions compromising gut barrier integrity and facilitating translocation of LPS and other endotoxins into systemic circulation (Chelakkot 2018 et al. 2018). LPS activates the Toll-like receptor 4 (TLR4) pathway, stimulating microglia and astrocytes to release pro-inflammatory cytokines including TNF-α, IL-1β, and IL-6 (Papageorgiou 2016, Ishijima and Nakajima 2021). Upon entering the circulation, LPS may cross a compromised blood–brain barrier, amplifying neuroinflammation and exacerbating oxidative stress (Matheoud 2019, Zhang 2022). Moreover, LPS-induced inflammation may promote the misfolding and aggregation of α-syn, further linking gut-derived immune activation to PD-related neurodegeneration. Together, these findings highlight LPS as a key microbial mediator linking gut dysbiosis to peripheral immune activation and central neurodegenerative processes in PD.

Bacterial amyloids—particularly curli fibers produced by *Escherichia coli*—have emerged as potential contributors to PD pathogenesis through cross-seeding, neuroinflammatory activation, and disruption of gut–brain communication (Schmit 2023). Curli fibers, primarily composed of the CsgA and CsgB subunits, share β-sheet–rich structures with pathological amyloids such as α-syn, enabling prion-like propagation via templated misfolding (Wang 2021a, Chapman 2002). Such structural mimicry allows curli fibers to accelerate the aggregation of endogenous amyloidogenic proteins, potentially contributing to a broad range of neurodegenerative disorders (Wang 2021a). In animal models, exposure to curli-producing *E. coli* enhances α-syn aggregation in both the enteric and central nervous systems, supporting a role in PD onset and progression (Chen 2016, Huynh 2023). This occurs via cross-seeding, whereby structural homology enables curli to nucleate α-syn misfolding and aggregation (Sampson 2020). These findings implicate bacterial amyloids such as curli in the broader landscape of protein misfolding disorders, including—but not limited to—PD.

Aberrant iron accumulation within specific regions of the brain is progressively implicated in contributing to the pathogenesis of PD (Riederer 1989, Genoud 2020, Li 2022) and various other neurodegenerative disorders (Yan and Zhang 2019, Ayton 2020, Damulina 2020). Neuroimaging and postmortem studies reveal elevated total iron and ferric iron (Fe³⁺) in the substantia nigra (SN) of PD patients (Sofic 1988), with SN iron load correlating with clinical disease severity (Li 2020a, Drayer 1986, Gorell 1995, He 2020, Cao 2022). Unbound ferrous iron (Fe²⁺) catalyzes the formation of reactive oxygen species (ROS) via Fenton chemistry (Myhre 2013, Trist 2018 et al. 2018), damaging lipids, proteins, and nucleic acids. Ferritin-bound iron normally buffers this activity, but with age, ferritin stores become saturated, and labile iron pools expand (Towe 1963 et al. 1963, Lowenstam 1981, Rouault 2013). The hydroxyl radical, a byproduct of iron-driven redox reactions, is among the most damaging ROS (Lyngsie 2018, Han 2019, Maher 2019), and has been associated with neuronal degeneration in PD (Fahn and Cohen 1992, Halliwell 2001). Elevated iron levels have been linked to α-syn aggregation (Chen 2019a), and α-syn overexpression itself may promote intracellular iron accumulation (Yarjanli 2017). Emerging studies also focus on nanoparticulate forms of iron, such as magnetite (Fe₃O_4_), which is detected in the SN of PD patients (Kirschvink 1992 et al. 1992, Dobson 2001). Containing both Fe²⁺ and Fe³⁺, its mixed-valence structure facilitates ROS generation. Biogenic, geometrically crystalline magnetite has been identified in the human brain, suggesting endogenous synthesis (Kirschvink 1992 et al. 1992, Yarjanli 2017, Vakili-Ghartavol 2020). Kirschvink et al. (Kirschvink 1992 et al. 1992) and Schultheiss-Grassi et al. (Schultheiss-Grassi 1999 et al. 1999) has observed the distinctive, geometric, highly-crystalline brain magnetite particles. In vitro data show that bare Fe₃O_4_ nanoparticles promote α-syn aggregation, whereas surface modifications, such as L-lysine coating, reduces this effect (Joshi 2015).

Hydrogen sulfide (H₂S) is a gaseous signaling molecule with both endogenous and microbial origins, predominantly generated by SRB (Murros 2022). Acting as a double-edged mediator, H₂S exerts neuromodulatory as well as neurotoxic effects depending on the physiological context (Hu 2010, Kida 2011). Several gut bacterial species capable of H₂S production have been implicated in PD, including *Helicobacter pylori, Clostridium difficile, Prevotella* spp., and *Desulfovibrio* spp. (Kimura 2011, Huang 2018, Kang 2020, Murros 2021, Huynh 2023). Beyond PD, excessive H₂S derived from SRB has also been implicated in inflammatory bowel disease and colorectal cancer (Kushkevych 2019b et al. 2019b, Kovac 2018 et al. 2018, Sun 2019, Dordevic 2021). Mechanistically, SRB not only produce H₂S but also compete with beneficial commensals for nutrients, thereby disrupting microbial balance and promoting inflammation (Kushkevych 2016, Coutinho 2017).

Owing to its high lipophilicity, H₂S readily diffuses across cell membranes independent of specific receptors, exerting broad biochemical and physiological actions (Mathai 2009). Within the nervous system, H₂S regulates synaptic plasticity, intracellular calcium homeostasis, and pH balance (Yong 2010, Kimura 2013, Liu 2014, Zhang 2020). At physiological concentrations (1.0–2.4 mM), it supports colonic epithelial health by enhancing mitochondrial respiration (Blachier 2019 et al. 2019). In murine models of PD, H₂S administration attenuates dopaminergic neuron loss and promotes neural stem cell proliferation in the subventricular zone (Wang 2021b). Additional protective effects include antioxidative stress mitigation (Tabassum 2020 et al. 2020, Scammahorn 2021), anti-inflammatory activity (Zhao 2019a, Li 2021a et al. 2021a) and inhibition of cellular respiration under pathological conditions (Fu 2012)—suggesting therapeutic potential for PD. However, excessive H₂S production can be detrimental (Kushkevych 2019a). It has been linked to gut inflammation, epithelial damage, and constipation—gastrointestinal symptoms that often precede motor dysfunction in PD (Dordevic 2021, Buret 2022, Murros 2022). Overgrowth of H₂S-producing bacteria may impair mucosal detoxification, reduce gut motility, and prolong exposure to toxic metabolites. Excess H₂S disrupts mitochondrial function, triggering cytochrome c release—a key event in α-syn oligomerization (Kushkevych 2019a, Zaorska 2020). Released cytochrome c acts as a peroxidase, promoting the formation of α-syn radicals and toxic aggregates, thereby inducing oxidative stress and dopaminergic neuron apoptosis (Hashimoto 1999, Kumar 2016 et al. 2016, Haque 2022). Misfolded α-syn can propagate from the enteric nervous system to the central nervous system via the vagus nerve, further amplifying neurodegeneration (Danyu 2019). Clinically, cerebrospinal fluid samples from PD patients reveal significantly elevated H₂S concentrations (5.48 ± 0.97 mg/L vs. 3.48 ± 1.68 mg/L in controls; p = 0.0004), supporting the notion that systemic H₂S accumulation may contribute to PD pathogenesis (Kushkevych 2019b et al. 2019b, Kovac 2018 et al. 2018, Sun 2019, Dordevic 2021). SRB can compete with beneficial commensals for nutrients, thereby disrupting microbial balance and promoting inflammation (Kushkevych 2016, Coutinho 2017). Excess H₂S may cross the blood–brain barrier and directly impair neuronal viability, enhancing α-syn aggregation (Scheperjans 2015, Huang 2018, Kang 2020, Murros 2021).

Bile acids (BAs), including primary bile acids—such as cholic acid, total muricholic acid, and β-muricholic acid—and secondary bile acids—such as tauroursodeoxycholic acid, taurohyodeoxycholic acid, and deoxycholic acid—are amphipathic molecules that function as biological detergents, facilitating the emulsification, solubilization, and absorption of dietary lipids and fat-soluble vitamins (Xiang 2021, Nie 2022). In PD, bile acid metabolism appears to be dysregulated. Specifically, elevated levels of gut microbiota-derived toxic secondary bile acids have been observed in PD patients (Li 2021b, Shao 2021). These altered bile acid profiles may contribute to disease progression by promoting α-syn misfolding and aggregation, a hallmark of PD pathology (Kaur 2024 et al. 2024). Comprehensive metabolomic profiling of plasma from PD patients further supports this notion, revealing significantly elevated bile acid levels compared to healthy controls (Shao 2021). These findings suggest that bile acids—once considered mere digestive molecules—may act as neuroactive metabolites that link gut microbial alterations to PD pathogenesis, offering new directions for biomarker discovery and therapeutic intervention.

### Potential mechanistic roles of gut microbial dysbiosis in PD pathogenesis

Longitudinal evidence further supports the stability of PD-associated dysbiosis. A two-year follow-up study demonstrated that gut microbiota differences between PD patients and controls remained stable over time (Aho 2019). The specific mechanisms by which microbiota alterations drive PD are not fully understood, as most evidence stems from preclinical studies and correlative human evidence (Braak 2006, Wang 2025). A central conceptual framework in this field is the microbiota–gut–brain axis (MGBA)—a bidirectional communication network that integrates neural, immune, and endocrine pathways to mediate interactions between the gut microbiota, enteric nervous system, autonomic nervous system, and central nervous system (Wang 2023b et al. 2023b). MGBA maintains intestinal and neurological homeostasis by modulating gut motility, barrier integrity, immune tone, and neurotransmission. In PD, microbial dysbiosis is thought to disturb this balance, triggering gut inflammation and increasing intestinal permeability and local misfolding of α-syn within the ENS. These pathophysiological changes may spread via the vagus nerve or systemic circulation to the CNS, contributing to microglial activation and dopaminergic neurodegeneration (Wang 2023b et al. 2023b, Aburto and Cryan 2024, Loh 2024). Microbial metabolites, including SCFAs, bile acids, lipopolysaccharides and hydrogen sulfide, are increasingly recognized as key mediators of this axis (Silva 2020 et al. 2020, Schneider 2024), microbial reconstitution or SCFA supplementation restores microglial immune competence (Erny 2015). These molecules can act locally or reach the brain via systemic routes or afferent neural fibers, modulating inflammation, synaptic function, and neuronal survival (Grabrucker 2023, Jiang 2025). Elucidating how gut microbiota and their metabolic outputs reshape host physiology via the MGBA is critical for advancing our understanding of PD pathogenesis and developing microbiota-targeted interventions (Needham 2022).

Gut dysbiosis and its associated metabolic alterations rise either through microbial metabolites or direct interactions with the host, the downstream effects converge on three interrelated mechanisms: immune activation, α-syn aggregation, and neurotransmitter dysregulation. Immune activation and neuroinflammation. Persistent neuroinflammation is a cardinal feature of PD and is mechanistically linked to gut microbiota alterations. Microbiota-derived signals can dysregulate both peripheral and central immune homeostasis through multiple, often synergistic, mechanisms. First, increased intestinal permeability (“leaky gut”) facilitates the translocation of microbial products, including LPS, into the systemic circulation. Elevated serum LPS levels correlate with increased pro-inflammatory cytokines in PD patients, reinforcing the link between gut barrier failure and systemic inflammation (Min 2022, d’Angelo 2023). Second, these circulating microbial components and inflammatory cytokines (e.g. TNF-α, IL-6, IL-1β) can cross the blood–brain barrier and activating microglia in the substantia nigra, contributing to oxidative stress, dopaminergic neuron loss, and disease progression (Koziorowski 2012, Qin 2016). Foundational experimental work has shown that germ-free mice display immature, hypo-responsive microglia, whereas recolonization with complex microbiota or supplementation with SCFAs restores microglial maturation and innate immune competence, underscoring a critical role for gut microbes and their metabolites in tuning CNS immune tone (Erny 2015). However, this relationship is highly context dependent. Seminal work in α-synuclein–overexpressing mice demonstrated that gut microbiota are required for motor deficits and neuroinflammation, and that administration of specific microbial metabolites, such as SCFAs, can promote microglial activation, pro-inflammatory cytokine production, α-syn aggregation, and motor deficits in a genetically susceptible host (Sampson 2016). Third, beyond generalized inflammation, a mitochondria-specific adaptive immune pathway linking the gut to PD-like neurodegeneration. In Pink1-knockout mice, intestinal infection with gram-negative bacteria induces mitochondrial antigen presentation and drives the expansion of cytotoxic CD8⁺ T cells that infiltrate the brain and damage dopaminergic projections, causing levodopa (L-DOPA) responsive motor impairment (Matheoud 2019). This work establishes a direct causal link between gut infection, genetic susceptibility, and autoimmune neurodegeneration. Converging evidence indicates that α-syn pathology can be initiated in the gut and ascend to the brain via the vagus nerve, providing a mechanistic bridge between enteric disturbances and central neurodegeneration in PD (Wu 2025a). At the intestinal level, specific microbes have been shown to trigger α-syn misfolding *in vivo*. In LRRK2-variant mice, curli-producing *Escherichia coli* induces pathological α-syn accumulation in the colon and brain through impaired autophagic clearance and curli-enriched extracellular vesicles, illustrating a gene–environment interaction centered on bacterial amyloids (Liang 2023). Strain-resolved work on sulfate-reducing *Desulfovibrio* spp. similarly shows that PD-associated isolates, drive robust α-syn aggregation, oxidative stress, and reduced survival in a *C. elegans* PD model (Mohammadi 2025 et al. 2025). Once formed in the gut, pathological α-syn can propagate retrogradely along the vagus nerve (Braak 2003). Intragastric delivery of α-syn fibrils leads to their sequential appearance in the dorsal motor nucleus of the vagus and brainstem nuclei. Truncal vagotomy completely abolishes this transmission and prevents neurodegeneration, establishing the vagus nerve as an essential conduit for gut-to-brain spread (Kim 2019a). Neural circuit–mapping further supports this route, as optogenetic activation of gut-innervating vagal sensory neurons has been shown to drive dopamine release in the substantia nigra via a parabrachial relay (Han 2018), providing anatomical substrate for long-range gut influences on nigrostriatal circuits. After entering the brainstem, α-syn pathology can disseminate transneuronally to vulnerable dopaminergic neurons, where it perturbs mitochondrial function, enhances oxidative stress, and disrupts synaptic signaling, in line with Braak’s staging model of a stepwise ascent from peripheral to central structures (Braak 2003, Braak 2004). Among neurotransmitter-related mechanisms, the most direct and clinically validated pathway is microbial metabolism of levodopa. Multiple studies now demonstrate that specific gut bacteria convert L-DOPA into dopamine in the small intestine, preventing the drug from reaching the brain and directly undermining therapeutic efficacy. Maini Rekdal et al. identified a defined biochemical pathway in *Enterococcus faecalis* converts orally administered L-DOPA into dopamine in the gut lumen before it can reach the brain (Maini Rekdal 2019). This microbial “drug hijacking” provides a mechanistic explanation for the pronounced individual variability and “on–off” fluctuations in treatment response. Van Kessel et al. showed that gut bacterial tyrosine decarboxylase (TDC), expressed by *Enterococcus faecalis*, converts levodopa to dopamine and escapes inhibition by carbidopa. Increased TDC abundance was associated with compromised levodopa levels and higher levodopa/carbidopa dosing requirements, with stool TDC gene abundance correlating with dosage (van Kessel 2019).

Collectively, these findings support a conceptual framework in which the MGBA mediates PD pathogenesis through a multistage process that links peripheral microbial disturbances to central nervous system pathology. We present the following pathogenetic model (Fig.. 4), in which gut microbial dysbiosis, barrier dysfunction, immune activation, and neuroinflammation are integrated into a stepwise cascade. In the healthy gut, commensal microbes produce SCFAs and vitamins that maintain epithelial integrity and immune homeostasis. By contrast, dysbiosis disrupts this equilibrium, leading to the accumulation of microbial-derived toxins, including lipopolysaccharide, curli amyloids, and hydrogen sulfide. These factors compromise epithelial junctions, increase intestinal permeability, and facilitate the translocation of microbial products into systemic circulation. Circulating pro-inflammatory signals sustain chronic immune activation, while α-synuclein aggregates form in submucosal neurons—potentially via cross-seeding by bacterial amyloids—and propagate retrogradely along the vagus nerve to the dorsal motor nucleus of the brainstem. In parallel, systemic inflammation and microbial products may impair blood–brain barrier integrity, enabling cytokines and endotoxins to access the CNS. These convergent processes activate microglia and astrocytes, establish a neuroinflammatory milieu, and ultimately exacerbate α-synuclein aggregation and dopaminergic neurodegeneration in the substantia nigra. Conversely, beneficial microbial metabolites such as SCFAs and vitamins reinforce barrier function and suppress inflammation, underscoring the therapeutic potential of microbiota-targeted interventions in PD prevention and treatment.

**Figure 4:**
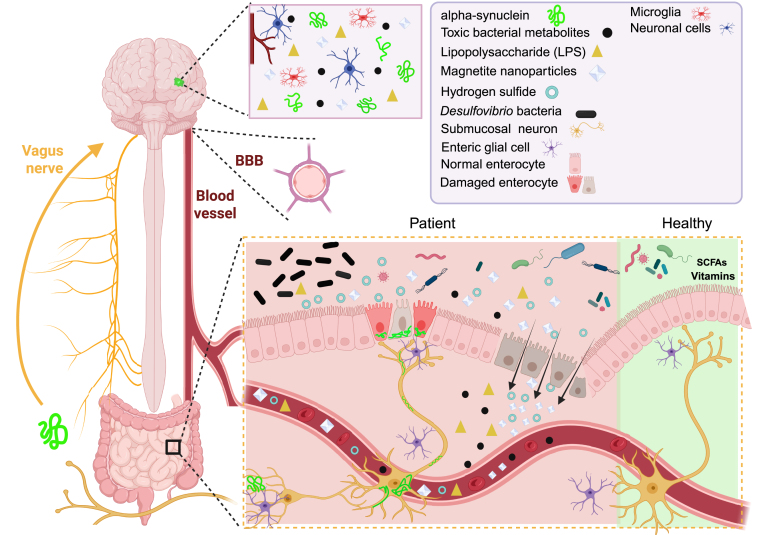
A proposed mechanism linking gut microbial dysbiosis to α-syn pathology and neurodegeneration in PD. The epithelial barrier integrity and immune homeostasis in healthy gut. Dysbiosis disrupts this balance and results in a leaky gut in patient. This figure was created with BioRender.com under an institutional license provided by the University of Helsinki.

### Potential therapeutic strategies for Parkinson’s disease

As a progressive neurodegenerative disorder, PD is characterized by a gradual onset and relentless deterioration of neuronal integrity. Accordingly, therapeutic strategies must extend beyond transient symptomatic relief and prioritize long-term interventions that decelerate disease progression, preserve dopaminergic function, and restore compromised neural circuits. Although invasive approaches such as deep brain stimulation alleviate motor symptoms, the greatest promise lies in disease-modifying therapies that provide neuroprotection, enhance neuronal resilience, and target core pathogenic processes (Loh 2024). In this section, we highlight three therapeutic avenues—antibiotics, probiotics, and fecal microbiota transplantation (FMT)—that exemplify increasing interest in targeting the gut–brain axis to influence PD pathogenesis and alter disease trajectory.

There is growing interest in the ancillary effects of antibiotics on gut microbiota and their potential to protect against dopaminergic neuron loss and motor impairments in PD (Cui 2023), particularly given the neuroactive and neurotoxic properties exhibited by many antibiotic classes (Rothstein 2005, Champagne-Jorgensen 2019, Negatu 2020, Hurkacz 2021 et al. 2021), highlighting their potential to mitigate PD-related pathologies such as α-syn misfolding, mitochondrial dysfunction (Chen 2010), dopaminergic cell death, and neuroinflammation (Jing 2014). The β-lactam antibiotic ceftriaxone has emerged as a candidate for neurodegenerative disease treatment due to its ability to cross the blood–brain barrier. Ceftriaxone demonstrates strong binding affinity to α-syn and effectively inhibits its polymerization *in vitro* (Ruzza 2014). In a 6-hydroxydopamine (6-OHDA) rodent model of PD, treatment with non-absorbable broad-spectrum antibiotics significantly attenuated dopaminergic neuron loss and motor deficits. Moreover, antibiotic administration suppressed the upregulation of pro-inflammatory markers including COX2, IL-1β, and TNFα (Reglodi 2017, Koutzoumis 2020). In MPTP-induced PD mouse models, ceftriaxone exerted neuroprotective effects, potentially via modulation of gut microbiota and suppression of neuroinflammatory responses (Rothstein 2005). The treatment improved behavioral and neuropathological outcomes, reduced astroglial and microglial activation—as indicated by decreased GFAP and Iba1 expression—and attenuated neuroinflammatory signaling (Zhou 2021). However, long-term exposure to antibiotics has been associated with an increased risk of PD (Kim 2025). Similarly, antifungal medications have been linked to PD onset within 1–10 years, based on a Finnish cohort study involving 13 976 PD cases and 40 697 controls (Mertsalmi 2020 et al. 2020). Comparative analyses across 30 EU countries have shown a positive correlation between increased PD prevalence and the use of narrow-spectrum penicillins and penicillinase-resistant penicillins (Ternák 2020 et al. 2020, Ternak 2022). Antibiotic use is a strong environmental determinant of gut microbiota composition and diversity. Repeated or prolonged exposure can disrupt microbial balance, potentially increasing susceptibility to PD (Frohlich 2016, Bicknell 2024, Radisavljevic 2024). Thus, although certain antibiotics may exert neuroprotective effects, prolonged administration of broad-spectrum agents is largely detrimental. By indiscriminately depleting both harmful and beneficial microbes, antibiotics can exacerbate dysbiosis, accelerate PD progression, and promote antimicrobial resistance. These drawbacks limit their potential as a sustainable therapeutic strategy for PD.

Probiotics are defined as live microorganisms that, when administered in sufficient amounts, confer health benefits to the host (Gu 2022, Ji 2023). The health benefit is induced mainly by restoring gut microbial balance, enhancing mucosal barrier integrity, and modulating immune responses (Hill 2014, Wang 2016, Zhao 2021). Manipulation of the gut microbiome has been found to alter emotional and cognitive behavior (Lee 2020, Chen 2021), neurotransmitter expression (Dicks 2022), and brain function in rodents (Cryan and Dinan 2012). Extensive preclinical and animal studies highlight the potential of probiotics to prevent and ameliorate gastrointestinal and central nervous system disorders (Magistrelli 2019, Xie and Prasad 2020, Leta 2021, Le Morvan de Sequeira 2022). Commonly used probiotic genera include the *Bifidobacterium* (e.g. *B. longum, B. breve*, and *B. infantis*) (Groeger 2013, Yao 2021) and lactobacilli (e.g. *Lactobacillus helveticus*, and *Lacticaseibacillus rhamnosus*) (Toscano 2017, Shi 2020). Probiotics are increasingly recognized as promising adjuncts for PD prevention and treatment. They suppress pathogenic microbial growth, alleviate gastrointestinal dysfunction (e.g. constipation, dysbiosis), and support the repair of damaged enterocytes, mucosal surfaces, microglia, and neurons.

An *in vitro* study demonstrated that peripheral blood mononuclear cells isolated from 40 PD patients and matched controls, when co-cultured with selected probiotic strains, exhibited significantly reduced production of pro-inflammatory cytokines (TNF-α, IL-6, IL-17A) and increased levels of anti-inflammatory cytokines (IL-4, IL-10), along with decreased ROS generation in both groups (Magistrelli 2019). Probiotics also restored cell membrane integrity and strongly inhibited the growth of *E. coli* and *K. pneumoniae* (Magistrelli 2019). In a PD mouse model, 24 weeks of probiotic administration conferred neuroprotective effects on dopaminergic neurons and mitigated motor impairments, improving gait patterns, balance, and motor coordination. Moreover, tyrosine hydroxylase (TH)-positive dopaminergic neurons in the substantia nigra were significantly preserved in probiotic-treated mice (Hsieh 2020). *Clostridium butyricum*, a commensal bacterium present in the gut of ∼20% of adults, exerted beneficial effects through butyrate production and suppression of pathogenic bacteria (Stoeva 2021). Oral administration of *C. butyricum* improved motor deficits attenuated dopaminergic neuron loss, synaptic dysfunction, and microglial activation in MPTP-induced PD mice. It also preserved gut barrier integrity by upregulating tight junction-associated proteins (Liu 2020, Sun 2021). In a 12-week clinical trial, probiotic supplementation improved scores on the Movement Disorder Society-Unified Parkinson’s Disease Rating Scale (MDS-UPDRS) and enhanced insulin metabolism (Tamtaji 2019). These findings suggest that probiotics exert neuroprotective effects by modulating the gut microenvironment, promoting anti-inflammatory cytokine production, and restoring intestinal homeostasis, highlighting their promise as adjunctive interventions for neurodegenerative diseases such as PD.

Despite prior research indicating the auxiliary and therapeutic efficacy of probiotics in PD, there are limitations. First, the long-term effectiveness of probiotic supplementation is unknown. It is unclear if administered strains can persistently colonize the gut microbiota in individuals with PD. Second, optimal dosing regimens, treatment duration, and strain-specific effects require further clarification. Although probiotics are generally considered safe, excessive or inappropriate use may lead to unintended consequences. In rare cases, certain strains may act as opportunistic pathogens, particularly in immunocompromised individuals, underscoring the importance of rigorous safety evaluation. Third, individual variability in host–microbiota interactions may influence the efficacy of probiotic interventions. Factors such as baseline microbial composition, host genetics, diet, and immune status can modulate both colonization resistance and therapeutic response, potentially leading to heterogeneous outcomes across patients. As such, personalized probiotic strategies—guided by microbiome profiling—may be required to achieve consistent benefits in PD.

Fecal microbiota transplantation (FMT), a practice with over six decades of clinical use, is an established therapy for recurrent *Clostridioides difficile* infection (Borody and Khoruts 2011, Ooijevaar 2019, Yadegar 2023). It entails the transfer of gut microbiota from a healthy donor to a recipient, with the goal of re-establishing microbial composition and functional equilibrium in the gut ecosystem (Gupta and Khanna 2017, Yadegar 2023). As a rapidly advancing microbiome-based intervention, FMT shows promise in correcting dysbiosis and restoring microbial homeostasis across multiple disease states, including inflammatory bowel disease (Wang 2014), ulcerative colitis (Moayyedi 2015, Ding 2019), irritable bowel syndrome (Holvoet 2021) and obesity (Zhu 2024). More recently, FMT has gained attention as a potential therapy for alleviating gut dysbiosis and associated clinical symptoms in PD (Xue 2020, DuPont 2023, Scheperjans 2024).

A preliminary clinical study in PD patients reported that colonoscopy FMT was well tolerated and improved both motor and non-motor symptoms—with sustained benefits lasting up to six months (Segal 2021). Case reports showed that FMT alleviated constipation and improved overall symptom burden in PD patients (Huang 2019, Kuai 2021, DuPont 2023). In one case, FMT led to sustained improvements in bowel function and nearly complete resolution of leg tremors within a week; however partial symptom recurrence was noted after two months (Huang 2019, Kuai 2021). A recent randomized trial demonstrated that oral FMT capsules successfully reconstituted gut microbial composition, leading to improved gastrointestinal symptoms and enhanced quality of life compared to placebo. The intervention also yielded a significant reduction in MDS-UPDRS total scores, suggesting a measurable clinical benefit (Cheng 2023).

In animal models of PD, FMT effectively mitigated gut microbial dysbiosis, reduced fecal SCFA levels, and alleviated motor impairments (Sun 2018, Zhao 2021). FMT also significantly elevated striatal levels of dopamine (DA) and serotonin (5-HT), indicative of neuroprotective activity. FMT attenuated neuroinflammation by suppressing microglial and astrocyte activation in the substantia nigra, downregulating key components of the TLR4/TNF-α signaling pathway, and inhibiting α-syn expression in both the gut and brain (Zhong 2021). These findings indicate that FMT exerts protective effects in PD by modulating gut-brain axis interactions, reducing neuroinflammation, and inhibiting TLR4/TNF-α-mediated immune responses (Sun 2018, Bruggeman 2024). Moreover, recipient mice displayed enhanced metabolic profiles and significantly lower fecal SCFA concentrations, indicating a broader systemic impact of FMT on neuroprotection and metabolic regulation (Tao 2023). Transplantation of fecal material from treated mice effectively alleviated motor impairments and preserved dopaminergic neurons in antibiotic-preconditioned PD models (Jiang 2023). Similarly, FMT from healthy human donors restored gut microbial balance and mitigated neurodegeneration in MPTP-induced PD mouse models. These therapeutic effects are attributed to reduced microgliosis and astrogliosis, mitigation of mitochondrial dysfunction through AMPK/SOD2 pathway activation, and restoration of nigrostriatal pericytes and blood–brain barrier integrity (Xie 2023). FMT offers a promising therapeutic avenue for patients with PD, particularly those with gastrointestinal comorbidities; however, its efficacy and safety need to be further investigated. Robust, large-scale randomized controlled trials are needed to confirm its clinical utility and evaluate long-term outcomes. Standardized protocols—including criteria for donor selection, dosing, and route of administration—are essential to ensure reproducibility and optimize clinical outcomes. Further mechanistic studies are warranted to delineate how FMT influences gut–brain communication, particularly its modulation of neuroinflammation, microbial metabolite production, and α-syn pathology.

Beyond established microbiota-targeted therapies, additional interventions have shown potential in attenuating PD-related pathology while concurrently reshaping gut microbial communities. The ketogenic diet (KD), a low-carbohydrate, high-fat dietary regimen, has demonstrated neuroprotective potential in neurodegenerative diseases. In MPTP-induced PD models, KD ameliorated motor deficits, preserved dopaminergic neurons, and decreased neuroinflammation, potentially through modulation of the gut–microbiota–brain axis (Jiang 2023). Salidroside, a phenylpropanoid glycoside derived from the plant *Rhodiola rosea*, exhibits potent antioxidant activity and neuroprotective potential. Neuroprotection appears to be mediated by suppression of ROS and nitric oxide (NO) production (Sheng 2013). In MPTP/MPP⁺ models, Sal pretreatment confers dose-dependent neuroprotection by limiting oxidative stress, modulating apoptotic signaling (Bcl-2/Bax), and suppressing α-syn aggregation (Wang 2015). Tetramethylpyrazine (TMP), a bioactive compound derived from traditional Chinese medicine, is widely utilized in the treatment of neurovascular and cardiovascular diseases (Meng 2021 et al. 2021). Preclinical studies suggest that TMP exerts neuroprotective effects, particularly in models of ischemic brain injury, by mitigating neuronal damage, reducing oxidative stress, and modulating inflammatory responses (Kao 2006). The compound will facilitate neurogenesis and functional recovery by promoting neuronal proliferation and differentiation (Xiao 2010, Wu 2013). In addition to dietary and phytochemical interventions, coffee consumption has been associated with a reduced risk of PD. In a large cohort study, individuals with high coffee intake had up to a 40% reduced risk of developing PD compared to non-consumers (Zhao 2024). Coffee contains numerous bioactive compounds that may contribute to its neuroprotective effects. Eicosanoyl-5-hydroxytryptamide (EHT), a coffee-derived compound, attenuates α-syn pathology in transgenic models by inhibiting protein aggregation (Lee 2011). EHT also exerts anti-inflammatory activity by inhibiting LPS-induced NF-κB signaling, iNOS expression, and NO synthesis (Lee 2013). These strategies not only mitigate neurodegeneration but also promote microbial homeostasis, underscoring the pivotal role of gut–brain axis disruption in PD pathogenesis.

Together, these findings highlight the therapeutic potential of microbiota modulation, dietary strategies, and bioactive compounds in PD. Targeting oxidative stress, neuroinflammation, mitochondrial dysfunction, and protein misfolding, they provide multi-pronged neuroprotection. Robust clinical trials are needed to confirm efficacy, define optimal regimens, and clarify underlying mechanisms. Future integration of these strategies into personalized treatment paradigms may enhance the therapeutic landscape of PD.

## Conclusion and future perspectives

Deciphering Parkinson’s disease increasingly requires an integrated framework that links specific gut microbes, their metabolites, and host neurophysiology into a coherent mechanistic model. While early research relied largely on descriptive associations, the field is now transitioning toward causal inference. As illustrated by key mechanistic studies, PD-relevant pathology is driven not by broad taxonomic imbalances but by discrete microbial functions—such as bacterial amyloid cross-seeding, vagal retrograde transport, metabolite-dependent microglial modulation, and pharmacomicrobiomic interference with L-DOPA therapy. These findings collectively indicate that gut microbes and their biochemical activities are not passive correlates but active contributors to neuroinflammation, α-synuclein misfolding, and symptom progression, helping to clarify the long-standing “chicken-or-egg” dilemma.

Bridging macro-level microbiome profiling with clinically meaningful biology now requires a focus on specific microbial taxa and their functional outputs. Pathobionts such as curli-producing *Escherichia coli* and H₂S-generating *Desulfovibrio* spp. have emerged as direct instigators of α-syn pathology, oxidative stress, and mucosal inflammation. Likewise, microbial metabolites, including short-chain fatty acids, lipopolysaccharides, curli fibers, and bile acids, have been frequently associated with PD progression. Importantly, SCFAs and hydrogen sulfide exhibit concentration-dependent duality in PD, functioning as either neuroprotective or neurotoxic agents depending on host context. At physiological levels, these compounds may confer neuroprotection, whereas elevated concentrations have been linked to neuroinflammation and cytotoxicity. Defining precise concentration thresholds remains challenging, as *in vivo* quantification and modulation of these metabolites is technically difficult in both clinical and preclinical contexts. Their pathological effects converge on shared mechanisms—oxidative stress, mitochondrial dysfunction, neuroinflammation, and α-syn aggregation. With increasing recognition of gut dysbiosis in PD pathogenesis, microbiota-directed therapies are emerging as putative therapeutic strategies. Among these, probiotics and fecal microbiota transplantation represent promising, sustainable approaches for restoring microbial balance and attenuating PD-associated pathology.

Future progress in PD research demands a decisive shift from descriptive cross-sectional profiling toward longitudinal characterization and mechanistic dissection. Integrating multi-omics approaches (metagenomics, metatranscriptomics, metabolomics) with immunophenotyping and neural circuit mapping will be critical for defining how specific microbial functions converge on core pathogenic pathways. Ultimately, translating mechanistic insights into clinically actionable biomarkers and targeted interventions will be essential for realizing microbiome-based strategies as credible therapeutic options in PD. From a sustainability perspective, microbiota-directed interventions—ranging from precision probiotics and engineered microbial consortia to fecal microbiota transplantation—offer a paradigm-shifting alternative. By leveraging native microbial ecology to restore physiological balance, these strategies align with sustainable healthcare goals and aim to provide durable, disease-modifying benefits with potentially fewer off-target effects than conventional chronic pharmacotherapy.

## Author contributions

Fang Chi (Conceptualization [equal], Writing—original draft [lead]), Kun Chen (Writing—review & editing [supporting]), Yue Yin (Writing—review & editing [supporting]), Janetta R. Hakovirta (Writing—review & editing [supporting]), Per Saris (Conceptualization [equal], Project administration [lead], Resources [lead], Supervision [lead], Validation [lead], Writing—review & editing [equal]).

## Data Availability

This article does not contain any new data. All data referred to are from published studies cited in the reference list.
